# A 45-Year-Old Woman With a Persistent Cavitary Consolidation

**DOI:** 10.1016/j.chpulm.2024.100104

**Published:** 2024-09-11

**Authors:** Anant Jain, Nao Hara, Marina Keller, Liying Han, Oleg Epelbaum

**Affiliations:** a Division of Pulmonary, Critical Care, and Sleep Medicine, Westchester Medical Center, Valhalla, NY; b Department of Pathology, Westchester Medical Center, Valhalla, NY; c Division of Infectious Diseases, Westchester Medical Center, Valhalla, NY

## Abstract

A 45-year-old woman with medical history significant only for benign breast disease presented to our institution in New York State with reports of nonproductive cough for 7 months as well as left chest and back pain for the preceding 2 weeks. There were no constitutional symptoms. She had never smoked and had immigrated to the United States from Guatemala 19 years earlier by traveling through Mexico and Arizona. There was no contact with anyone diagnosed with TB within the previous year, and no international travel during the same time period. She had not undergone any dental procedures and had not experienced syncope prior to the onset of symptoms.

On admission, the patient was afebrile and had normal vital signs, including oxygen saturation. General examination revealed a well-appearing woman with no signs of wasting and no digital clubbing. Lung auscultation was notable for decreased breath sounds over the left lower chest posteriorly. There were no other significant examination findings. Routine laboratory evaluation was remarkable only for a mildly elevated blood leukocyte count of 12,200/mm^3^ (normal range, 4,500-10,800/mm^3^). Testing for HIV was negative.

The patient’s admission chest radiograph (CXR) revealed an opacity with a large air-fluid level in the mid-left hemithorax interpreted by our radiologist as a hiatal hernia (Fig 1). However, a CXR performed 2 months earlier at another hospital had been officially read as normal. This prompted CT imaging of the chest, which showed an extensive cavitary consolidation involving nearly the entire left lower lobe (Fig 2). Several 3 to 4 mm pulmonary nodules were noted elsewhere in the lungs (not shown), central bronchi were patent, and there was no lymphadenopathy.

**Figure 1:**
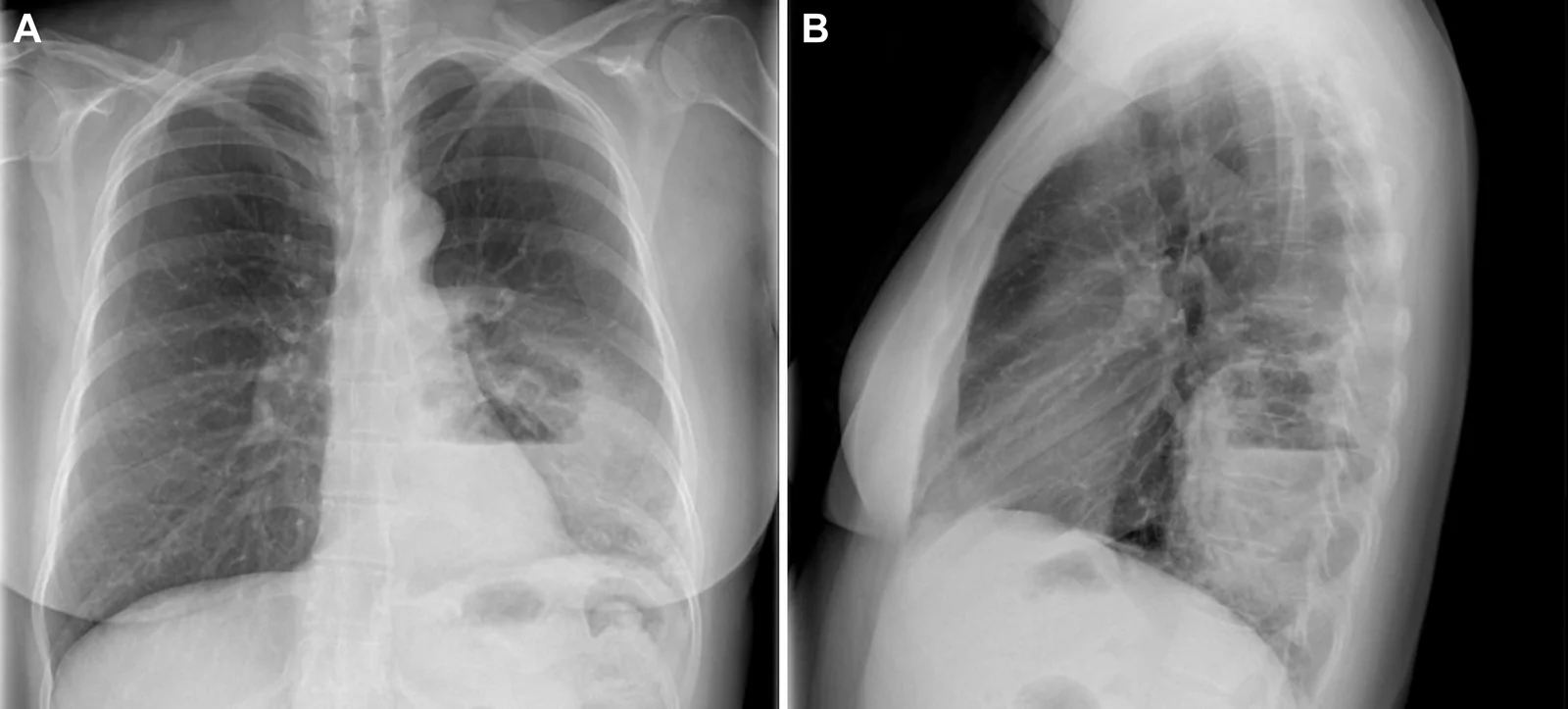
Patient’s frontal (A) and lateral (B) chest radiographs upon initial presentation to our hospital showing an opacity with an air-fluid level projecting over the region of the left lower lobe.

**Figure 2:**
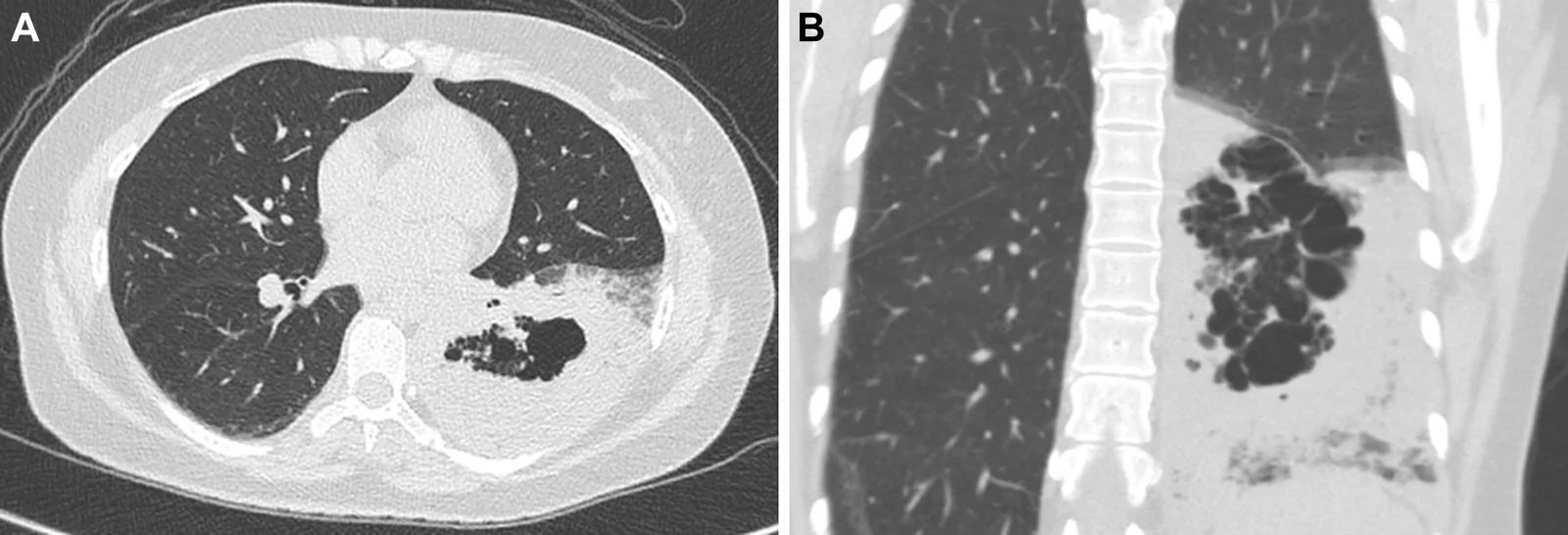
Representative axial (A) and coronal reconstruction (B) images from the patient’s CT scan of the chest set to lung window showing near-complete consolidation of the left lower lobe with an extensive cavitary component.

The constellation of findings in this case was favored to represent an infection with either polymicrobial lung abscess or *Mycobacterium tuberculosis/*nontuberculous mycobacteria or a fungal pathogen such as *Aspergillus* species. With that in mind, bronchoscopy with bronchoalveolar lavage and biopsy of the peripheral rim of the cavitary consolidation were performed by using fluoroscopic guidance only. Histopathology was nondiagnostic, showing just an interstitial lymphocytic infiltrate. Comprehensive culture-based and molecular diagnostics performed on bronchoalveolar lavage fluid were negative for mycobacterial and fungal etiologies. At that point, the patient’s IV antibiotics were converted to oral amoxicillin-clavulanic acid, and she was discharged with instructions to complete 4 additional weeks of treatment for presumed bacterial lung abscess. Upon completion of therapy, she returned to the hospital due to persistence of pain and cough, the latter now accompanied by copious tan-colored sputum production. Repeat chest CT imaging showed no improvement in the left lower lobe cavitary consolidation and possibly even its interval growth. An abdominal CT scan revealed no additional findings.

The primary diagnostic consideration remained infection refractory to administered antibiotic therapy. The patient was therefore referred for video-assisted thoracoscopic resection of the presumed left lower lobe abscess unresponsive to medical management. The lobectomy specimen showed an 8.0 × 7.5 × 3.5 cm necrotic cavitary lesion containing hemorrhagic mucus. The pathologic findings of the resected specimen are illustrated in Figure 3. Following surgery, the images from the reportedly normal CXR obtained 2 months prior to her initial presentation to our hospital were made available for review and are shown in Figure 4.

**Figure 3:**
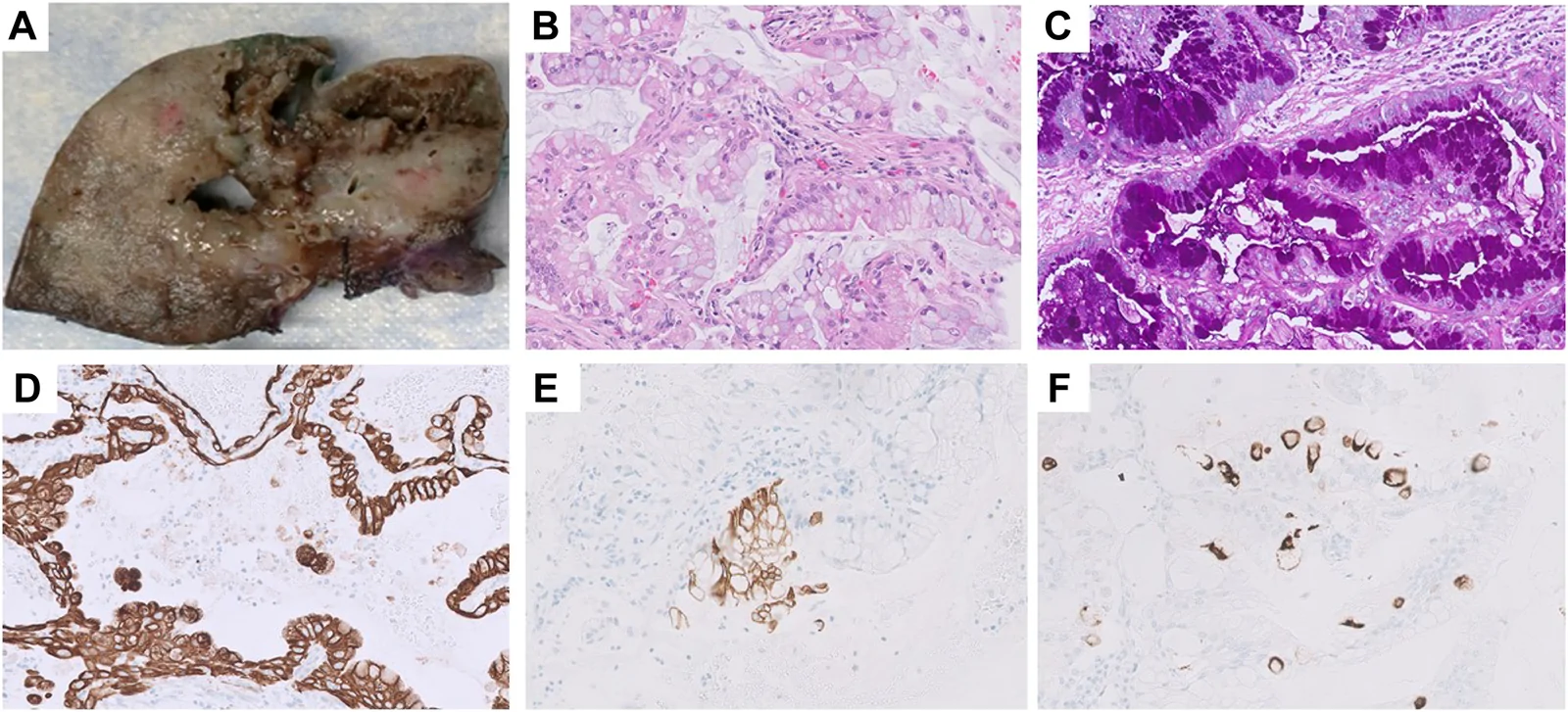
A, Gross appearance of the mucin-filled cavitary lesion located within the resected left lower lobe measuring 8 cm in greatest dimension. The cut surface of the formalin-fixed specimen exposes the heavily mucinous composition of the lesion. B, Microscopic section of the lesion stained with hematoxylin and eosin and viewed under high power (original magnification × 200) demonstrates replacement of normal lung parenchyma by nests of minimally atypical neoplastic cells and pools of extracytoplasmic mucin. C, The abnormal lung tissue stained positive with periodic acid-Schiff. Immunohistochemical staining was positive for CK7 (D), focally positive for CK20 (E), and showed scattered positivity for MUC2 (F). There was negative staining for thyroid transcription factor-1 and napsin A.

**Figure 4:**
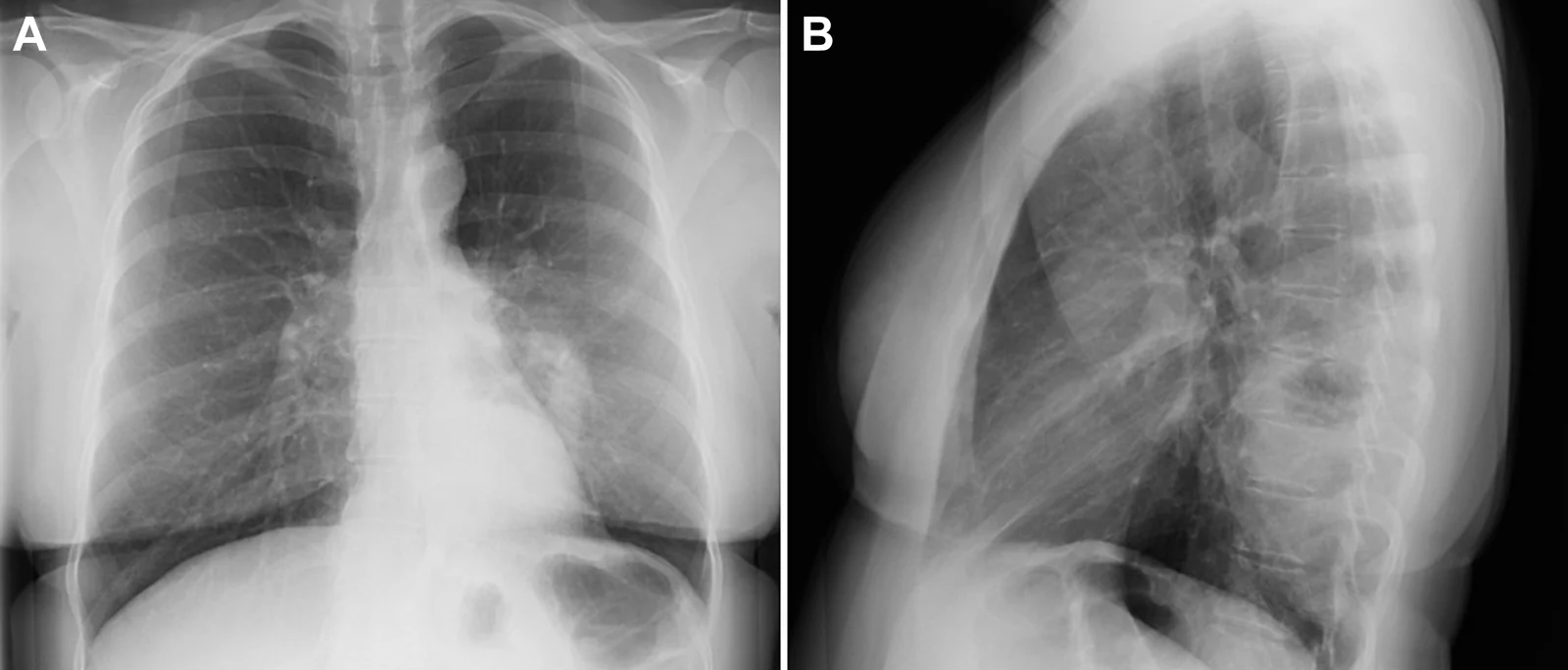
Patient’s frontal (A) and lateral (B) chest radiographs performed elsewhere 2 months prior to initial presentation to our hospital; this study was read as normal by the local radiologist. In hindsight, it is apparent that a subtle abnormality was already present in the region of the left hilum on the frontal view, projecting over the spine on the lateral view.

*What is the diagnosis?*

*Diagnosis:* Invasive mucinous adenocarcinoma of the lung

## Discussion

### Clinical Discussion

Invasive mucinous adenocarcinoma (IMA) is a subtype of primary lung adenocarcinoma formerly referred to as mucinous bronchoalveolar cell carcinoma and renamed in the 2015 update of the World Health Organization’s lung tumor classification schema.^1^ It comprises between 1.5% and 10% of resected lung adenocarcinomas depending on the geographic origin of the data set.^2^^,^^3^ Although IMA can occur in all patient types, its predilection for afflicting younger female individuals who have never smoked can falsely lower the clinical index of suspicion for primary lung cancer, as it did in the current case.

Mucus hypersecretion by neoplastic cells in these tumors accounts for the classical association of IMA with bronchorrhea. Copious sputum production can raise initial suspicion for infection and thus introduce further diagnostic delays. An additional challenge is the poor yield of bronchoscopic biopsy in IMA compared with non-mucinous lung adenocarcinoma (NMLA).^4^ This is likely due to the bronchoscopist’s inadvertent sampling of the mucin pool in which the already bland-appearing malignant cells of IMA are sparsely distributed.^5^ Compared with NMLA, IMA has greater propensity for local extension as opposed to nodal or distant spread, although the approach to mediastinal sampling at time of diagnosis remains the same. As a result of this tendency and relatively indolent growth, IMA often offers an opportunity for curative resection of what at the time of surgery is still only a locally advanced tumor. However, IMA carries a high risk of recurrence, which also tends to be limited to the lungs rather than taking the form of extrapulmonary metastases that are common in NMLA.^6^ Notably, patients with IMA and NMLA have been shown to have similar overall survival following resection.^7^

### Radiologic Discussion

The chest CT appearance of IMA is conventionally divided into 2 patterns: the more common nodular or mass-like pattern resembling conventional lung malignancy and the less common pneumonic-type pattern that can be indistinguishable from consolidated lung in the setting of infection. The pneumonic-type pattern is characteristic of IMA and is associated with excessive mucus production, making this combination a particularly misleading mimic of infection. The frequent lower lobe involvement in pneumonic-type IMA can lead to misdiagnosis as infection rather than primary lung cancer. The study patient exhibited all of these confounding factors. A noteworthy feature of the patient’s pneumonic-type lesion was its cavitation, which has been described only occasionally in cases of IMA and thus cannot be considered typical.^8^^,^^9^ The presence of a thick-walled cavity further complicated initial diagnostic reasoning. Although older studies that analyzed CXR characteristics of lung cavities showed a direct correlation between increasing wall thickness and malignancy,^10^ this relationship has not been as consistent in more contemporary CT imaging-based studies.^11^ Retrospective cohort studies of resected IMA cases have identified the pneumonic-type pattern as an adverse prognostic indicator with respect to both recurrence and survival.^12^^,^^13^

It has been recognized for decades that mucinous neoplasms in general are prone to false-negative results of fluorodeoxyglucose-PET (FDG-PET) scanning due to their low cellularity relative to the amount of associated mucin, which is a metabolically inert substance.^14^ The low sensitivity of FDG-PET has been described more recently in mucin-producing lung adenocarcinomas specifically, including IMA.^15^ The current patient did not undergo FDG-PET scanning as part of her evaluation, but a representative PET-CT image from another patient with confirmed IMA showing very patchy uptake is depicted in Figure 5. The ability of IMA to imitate other lung conditions such as infection with nontuberculous mycobacteria and pulmonary actinomycosis among others has been documented in a number of reports (Table 1).

**Figure 5:**
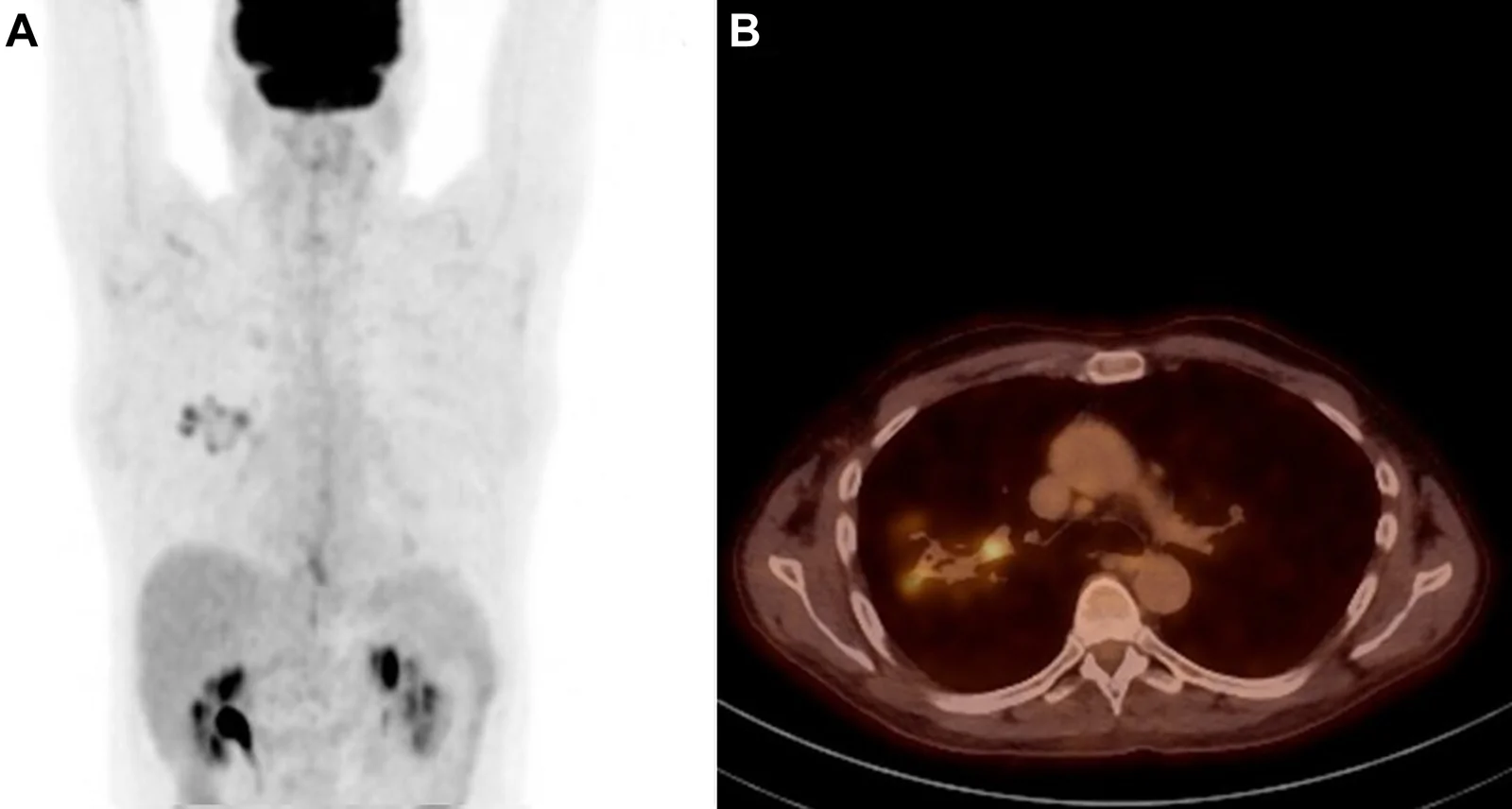
Whole-body maximum intensity projection image (A) and axial CT-fusion image (B) from a PET scan of another patient with proven invasive mucinous adenocarcinoma in the right lung highlighting the patchy ^18^fluorodeoxyglucose avidity of the malignant lesion.

**Table 1:** List of Published Reports of Misdiagnosis of Invasive Mucinous Adenocarcinoma as Other Lung Conditions

Condition	Author, Year
Non-tuberculous mycobacterial infection	Morichika et al, 2014^16^
Chronic eosinophilic pneumonia	Beom et al, 2014^17^
Pulmonary actinomycosis	Zhu et al, 2022^18^
Pulmonary echinococcosis	Alwatari et al, 2022^19^
ARDS	Kawasumi et al, 2023^20^
Community-acquired pneumonia	Yeung et al, 2023^21^

### Pathologic Discussion

IMA is characterized histologically by goblet or columnar neoplastic cells with basally oriented, bland-appearing nuclei and abundant intracytoplasmic and extracellular mucin. IMA is not the only subtype of lung adenocarcinoma characterized by abundant mucin: the same is true of its much rarer colloid and enteric variants. The renaming of IMA from mucinous bronchoalveolar cell carcinoma in the World Health Organization’s 2015 update was prompted by the recognition that an invasive component is invariably intermixed with the lepidic pattern of spread in these tumors and, in fact, can sometimes predominate as was the case in the current patient. If the extent of the invasive component measures < 5 mm in a resected lung nodule (ie, ≤ 3 cm) with mucinous morphology, such a lesion is then referred to as minimally invasive mucinous adenocarcinoma.

An alternative method of propagation, so-called spread through airspaces (STAS) or aerogenous metastasis, is defined by the presence of single cancer cells or clusters thereof in airspaces beyond the edge of the main tumor. STAS occurs in about 75% of IMA specimens, which is a much higher rate than that seen in NMLA as a whole, although the micropapillary and solid-predominant histology findings of NMLA also frequently exhibit STAS.^23^, ^24^, ^22^ It has been hypothesized that STAS may account for the tendency of IMA to recur elsewhere in the lungs following resection, particularly after sub-lobar resection in which STAS is an independent risk factor for recurrence.^25^ It has also been proposed that the mucus-filled airspaces of IMA are a particularly effective conduit for the distant travel of detached cancer cells by this mechanism.^24^

The immunohistochemical staining pattern of IMA contrasts with that of NMLA in important ways (Table 2). For example, in addition to the positivity of CK7, which both tumors share, IMA can be focally positive for CK20, a marker associated with hepatobiliary or mucinous gastrointestinal malignancy. Positivity of MUC2, another stain that identifies mucin-producing cells of IMA, is likewise not a feature of NMLA. Conversely, thyroid transcription factor-1 staining is absent in IMA and present in ≥ 75% of NMLA cases.^26^ In light of the overlap in immunohistochemistry between IMA of the lung and metastatic adenocarcinoma of abdominal origin, differentiating between the 2 can benefit from correlation with history (ie, presence or absence of a known abdominal primary) and radiology, wherein a solitary pulmonary lesion and normal abdominal organs favor lung origin.

**Table 2:** Summary of Notable Differences Between IMA and NMLA

Parameter	IMA	NMLA
Clinical/radiology		
Proportion of female participants	Higher	Lower
Association with smoking	Weaker	Stronger
Bronchorrhea	More common	Less common
Pneumonic-type imaging	More common	Less common
Sensitivity of FDG-PET	Lower	Higher
Yield of small biopsy	Lower	Higher
Nodal involvement	Less common	More common
Typical recurrence pattern	Local	Systemic
Pathology/genetics		
STAS	More common	Less common
TTF-1 staining	Negative	Positive
CK20 staining	Focally positive	Negative
MUC2 staining	Positive	Negative
EGFR mutation	Less common	More common
KRAS mutation	More common (G12D/V)	Less common (G12C)

FDG-PET = fluorodeoxyglucose-PET; IMA = invasive mucinous adenocarcinoma; NMLA = non-mucinous lung adenocarcinoma; STAS = spread through air spaces; TTF-1 = thyroid transcription factor-1.

Differences between IMA and NMLA also extend to their respective molecular genetic profiles. EGFR mutations that are frequently encountered and therapeutically targeted in NMLA are almost invariably absent in IMA. On the other hand, although only about 25% of NMLA harbor a KRAS mutation, roughly the same percentage of IMA are negative for a KRAS mutation, making it very prevalent in the latter.^2^^,^^27^ Of note, 2 KRAS inhibitors, sotorasib and adagrasib, target the G12C KRAS mutation typically found in non-small cell lung cancer, whereas the KRAS mutations characteristic of IMA are G12D and G12V; these are not actionable with the current molecular armamentarium against non-small cell lung cancer. Table 2 summarizes the usual pathologic features of IMA and NMLA as well as lists key differences in their clinical and radiologic presentations.

The current study patient’s tumor exhibited the KRAS G12D mutation and was negative for Programmed death-ligand 1 (PD-L1). Resection margins were clear, and there was no visceral pleural invasion. She was pathologically staged as T4N0M0 or IIIA. The patient completed 4 cycles of adjuvant pemetrexed and carboplatin followed by immunotherapy with pembrolizumab. Subsequent to these treatments, 5 months after her initial diagnosis, the patient was admitted to the hospital for worsening dyspnea, at which point widespread disease recurrence in both lungs was diagnosed. After thorough discussions, the patient and her family elected to pursue comfort-oriented care, and she ultimately died of the cancer.

## Conclusions

IMA is a subtype of lung adenocarcinoma that is particularly susceptible to misdiagnosis as an infection due to the demographic (female individual who had never smoked), clinical (bronchorrhea), and imaging (lower lobe consolidation) characteristics that are frequently encountered in affected patients. In the current case, the presence of cavitation, which is not expected in IMA, further diverted diagnostic suspicion away from malignancy and toward lung abscess. There is no “magic formula” for maintaining a healthy index of suspicion for IMA when confronted with a presentation that is unusual for primary lung cancer. Hopefully, however, our experience will remind clinicians to consider IMA before anchoring on a benign diagnosis in similar cases.

## Financial/Nonfinancial Disclosures

None declared.
